# Ki-67 Expression in CRC Lymph Node Metastasis Does Not Predict Survival

**DOI:** 10.1155/2015/131685

**Published:** 2015-09-13

**Authors:** Sandra F. Martins, Ricardo Amorim, Sílvia Coelho Mota, Luís Costa, Fernando Pardal, Mesquita Rodrigues, Adhemar Longatto-Filho

**Affiliations:** ^1^Life and Health Sciences Research Institute (ICVS), School of Health Sciences, University of Minho, Campus of Gualtar, 4710-057 Braga, Portugal; ^2^ICVS/3B's-PT Government Associate Laboratory, Braga/Guimarães, Campus of Gualtar, 4710-057 Braga, Portugal; ^3^Surgery Department, Hospitalar Center Trás-os-Montes e Alto Douro, Unidade Hospitalar de Chaves, Avenida Dr. Francisco Sá Carneiro, 5400-279 Chaves, Portugal; ^4^Pathology Department, Braga Hospital, Sete Fontes, São Victor, 4710-243 Braga, Portugal; ^5^Coloproctology Unit, Braga Hospital, Sete Fontes, São Victor, 4710-243 Braga, Portugal; ^6^Laboratory of Medical Investigation (LIM) 14, Faculty of Medicine, University of Sao Paulo, 01246-903 São Paulo, SP, Brazil; ^7^Molecular Oncology Research Centre, 14784-400 Barretos, SP, Brazil

## Abstract

Colorectal cancer is one of the most common malignancies and a leading cause of cancer death worldwide. Molecular markers may improve clinicopathologic staging and provide a basis to guide novel therapeutic strategies which target specific tumour-associated molecules according to individual tumour biology; however, so far, no ideal molecular marker has been found to predict disease progression. We tested Ki-67 proliferation marker in primary and lymph node metastasis of CRC. We observed a statistical significant difference between the positive rates of neoplastic cells positively stained by Ki-67 in both sites, with remarkable increased number of Ki-67 positive cells in primary tumor cells compared to cancer cells that invaded lymph nodes. We can speculate that the metastatic CRC in lymph node can be more resistant to the drugs that target cellular division.

## 1. Introduction

Colorectal cancer (CRC) is one of the most common malignancies and a leading cause of cancer death worldwide [1–5]. European countries rank the highest in the global statistics, in terms of both CRC incidence and mortality [4, 6], although in recent years, a decline in CRC mortality rates has been observed, mostly due to improvement in earlier diagnosis and treatment [4, 7].

In Portugal, official data revealed that CRC is the second most common type of cancer, in both men and women, and in 2008 it was responsible for 18.7% and 15.1%, respectively, of all cancer in Portugal [8]. Regarding mortality, unlike European data [9], there was an average increase of 3% from 2000 to 2005 [10] and in 2012 incidence and mortality rates are higher than European rates [11].

For CRC, the pathologic clinical stage is currently the single most important prognostic factor [1–4, 12, 13], correlating with long-term survival [4, 14–17], although it does not fully predict individual clinical outcome [4, 17–19]. This is particularly true for those tumours with intermediate stage disease (T3-T4N0M0) [19], where one-third of patients with tumour-free lymph nodes have recurrences, and therefore adjuvant chemotherapy may be beneficial [20]. In this group, carcinoma cells are not detected in lymph nodes by conventional staging methods in 24% of patients. So, lymphatic staging is essential to improve treatment of these patients, indeed one-third of the patients submitted to curative intent surgery die of local and/or distant tumour recurrence [4, 15]. Abdominal lymph nodes (38%) are the second most frequent site of metastasis (38%), just after liver, that is, the organ most frequently involved (38–60% of cases) and followed by lung (38%) and peritoneum (28%) [4, 13].

A common feature of all cancers is the imbalance that exists between the proliferative activity and cell death; therefore, the evaluation of cell proliferation rate may be interesting in the study and characterization of tumours [21]. Some molecules, such as the Ki-67 protein, permit this assessment and are used as markers of proliferation because Ki67 expression is dependent of cell division rate; thus, overexpression of these markers may suggest a disruption in the proliferation mechanism leading to the appearance of tumours [21].

Ki-67 protein, when used to evaluate the percentage of dividing cells, allows us to determine neoplastic growth [21] and has been documented to correlate with neoplastic progression [22] showing different levels of expression between normal mucosa, adenoma, and adenocarcinoma [23], verifying a progressive increasing of positive Ki-67 expression from the first (normal mucosa) to the last part of the tissue (adenocarcinoma) [22, 24, 25].

Other studies correlate Ki-67 with the degree of malignancy, tumour invasiveness [25, 26], metastatic potential [21], patient survival, and the risk of relapse [27, 28]. Thus, a high Ki-67 expression in tumour cells is assumed to correlate with a poor tumour differentiation [24, 26] and an increased infiltration of the bowel wall (pT) [26]. Micev et al. [29] demonstrated that there is an association between Ki-67 expression and a less effective response in patients undergoing chemotherapy.

Other correlations with clinical and pathological data were also investigated and a correlation was detected between a high expression of this protein and the following variables: patient's age [25], tumour size [30], tumour localization [28], dysplasia degree [30], the presence of lymph node metastasis [22, 25, 28], and TNM [25] and Dukes [28] classification. Thus, the younger is the patient, the greater is the cellular proliferation and the lower is the degree of differentiation; with increasing malignancy a increased frequency of invasion and metastasis are observed and thus poorer prognosis [25].

In CRC, the analysis of colon adenomas has shown a different pattern for Ki-67 expression between normal tissue, adenomas, and adenocarcinomas, being limited to the crypts in normal tissue and expressed both in the crypts and in the surface epithelium in adenomatous polyps (tubular, villous) [31] and distributed homogeneously in adenocarcinoma [32]. Nussrat et al. [30] also observed an increase in Ki-67 rates being associated with the growth and rise of dysplasia in adenomas.

Studies on CRC indicated Ki-67 as a prognostic marker as the survival rate for patients with high expression of Ki-67 is significantly lower compared to those with low expression [25, 33–35] and a predictor of CRC recurrence [36]. Also significant associations were found between higher index of Ki-67 and increased tumour penetration [35, 37], the presence of lymph node [22, 35] and distant [35] metastasis, advanced TNM stage [32, 35], highest degree of differentiation, and subtypes of adenocarcinoma other than mucinous [38].

However, not all studies are in agreement, and no correlations were observed with patient age, gender, tumour location [22, 30, 33, 39], and the type of adenoma [30] for some of them. Allegra et al. [40] described inverse associations, with a lower rate of Ki-67 to be associated with greater recurrence and worse overall survival and Jansson and Sun [39] did not find any associations between index Ki-67 and clinicopathological data or prognosis.

Regarding the use of Ki-67 in CRC lymph node metastasis, no information is available, and the only similar study found compares Ki-67 index in primary tumour with peritoneal metastasis and had observed a lower proliferative index in metastasis compared with the primary tumour [41]. However, in other types of cancer, in particular breast cancer, a higher Ki-67 index was found in lymph node metastasis than in primary tumours [42–44], suggesting greater aggressiveness of these [42] and that the use of Ki-67 in lymph node metastasis may be important in selecting the appropriate treatment for certain subgroups of patients [45].

Therefore, given the limited information concerning Ki-67 index in CRC lymph nodes metastasis and primary tumour, this study becomes relevant to determine Ki-67 index in the primary tumour and, respectively, lymph nodes metastasis whilst trying to establish correlations with this and clinicopathological data and the patient's prognosis.

## 2. Materials and Methods

### 2.1. CRC Tumour Series

Tissue samples and data from 672 patients treated in Hospital de Braga, Portugal, between January 1, 2005, and January 1, 2010, with CRC diagnosis were collected prospectively. Tumour localization was recorded and classified as colon and rectum (between anal verge and 15 cm at rigid rectoscopy).

The histological type of CRC was classified by two experienced pathologists and tumour staging was graded according to the TNM classification, sixth edition [46]. Tissue microarrays (TMAs) were constructed with the CRC series of formalin-fixed, paraffin-embedded tissues and analyzed by immunohistochemistry. Prior to tumour construction, hematoxylin and eosin sections were reviewed to select representative areas of the tumour and normal-adjacent tissue. Each case was represented in the TMA by at least two cores of 0.6 mm.

### 2.2. Lymph Node Metastasis Series

From the same series of colorectal cancer, patients with the diagnosis of CRC lymph node metastasis were selected and a series of 210 patients were also collected.

Additionally, 35 patients, with the diagnosis of CRC but without lymph node metastasis, were also selected for control of protein lymph node expression (stages T1 and T2/N0).

TMAs were constructed with the lymph node metastasis series of formalin-fixed, paraffin-embedded tissues and analyzed by immunohistochemistry. Each case was represented in the TMA by at least two cores of 0.6 mm.

The study protocol was approved by the Ethics Committee of Hospital de Braga and ICVS.

### 2.3. Immunohistochemistry

CRC and lymph nodes TMAs protein expression was evaluated by immunohistochemistry. Detailed information is given in Table 1. After the immunohistochemical procedure, the slides were evaluated and then photographed under a microscope.

**Figure 1:**
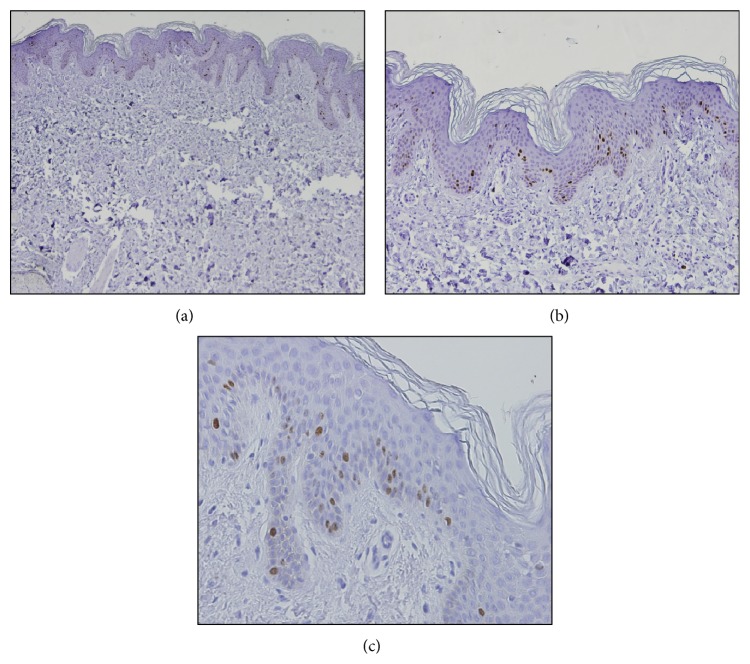
Immunohistochemical expression of Ki-67 in samples of skin: (a) original magnification ×40; (b) original magnification ×100; (c) original magnification ×200.

For positive control of the expression of Ki-67 a sample of the skin was used (Figure 1).

### 2.4. Immunohistochemical Evaluation

The percentage of immunoreactive cells was determined (which was named the Ki-67 index), counting a total of 100 cells per section at ×20 magnification, and each one was assigned a score from 0 to 3, as previously described by Pinheiro et al. [47].

Immunoreaction final score was defined as the sum of both parameters and grouped as negative (0-1) and positive (≥2). Evaluation of protein expression was performed by blind analysis by two observers and discordant cases were discussed in a double-head microscope in order to determine a final score.

### 2.5. Statistical Analysis

All data were analyzed using the Statistical Package for the Social Sciences, version 19.0 (SPSS Inc., Chicago, IL, USA). All comparisons were examined for statistical significance using Pearson's chi-square (*χ* ^2^) test and Fisher's exact test (when *n* < 5), with the threshold for significance *P* < 0.05. Survival curves were determined for overall survival by the Kaplan-Meier method and log-rank test.

Expression differences between lymph node metastasis and primary CRC were tested with McNemar test, with the threshold for significance *P* < 0.05.

## 3. Results

### 3.1. Ki-67 Expressions in CRC Samples

A total of 672 samples were organized into TMAs, including tumour and normal adjacent epithelium (NA^E^). Sections were evaluated for immunoexpression and the obtained results are given in Table 2, which summarizes the frequency of Ki-67 expression in tumour cells and NA^E^.

We observed that 68.2% (*n* = 345) of the samples of tumour tissue were positive for Ki-67, as compared to 24.3% (*n* = 34) of samples of the samples of NA^E^. Thus, it was concluded that the Ki-67 expression is significantly higher in tumour tissue (*P* < 0.05), such as is shown in Table 2.

**Figure 2:**
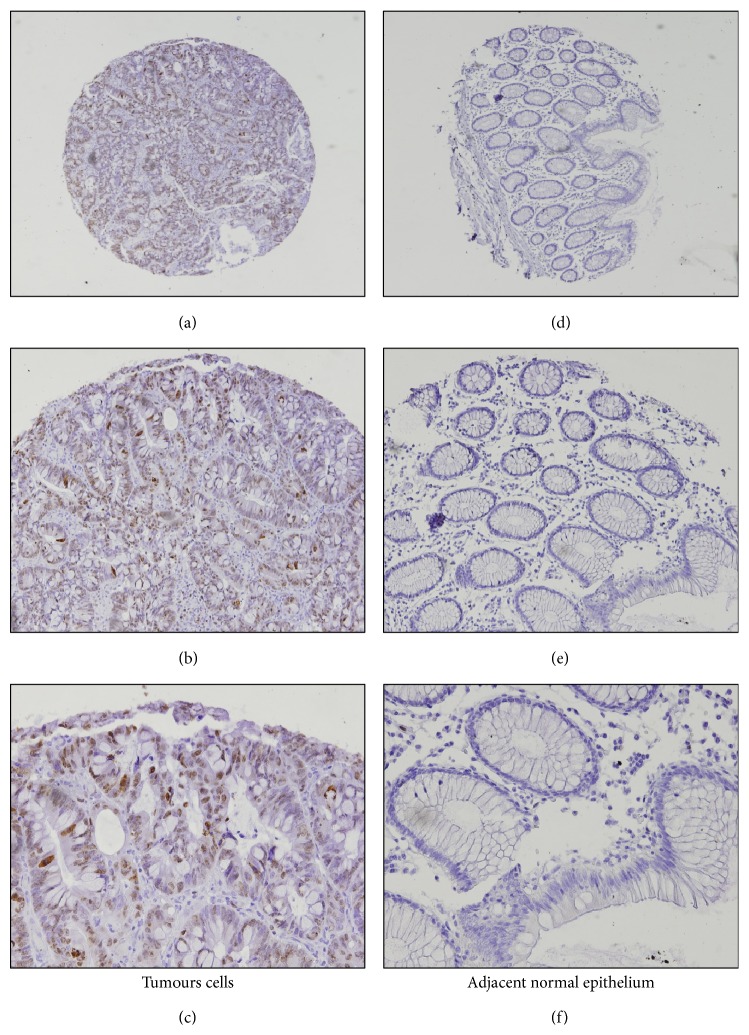
Immunohistochemical expression of Ki-67 in colorectal cancer samples: ((a) and (d)) original magnification ×40; ((b) and (e)) original magnification ×100; ((c) and (f)) original magnification ×200.

Figure 2 shows representative cases of positive staining for Ki-67 in tumour cells and in NA^E^.

### 3.2. Associations between Ki-67 Expressions in CRC Tissues and Clinicopathological Data

The associations observed between the expression of Ki-67 in CRC and the clinicopathological data are described in Tables 3 and 4.

Analyzing the results in these tables, we found an association between the expression of Ki-67 and “tumour penetration” (*P* = 0.013) and “tumour differentiation” (*P* = 0.049).

For “tumour penetration,” we observed a decreasing expression of Ki-67 from the pT1 (79.3%) to pT3 (68.1%) tumours and then a rise in expression for adenocarcinoma with invasion of other organs or structures (pT4) (73.1%).

Regarding “tumour differentiation,” we observed an increasing expression of Ki-67 from the well-differentiated to the undifferentiated tumours, namely, well differentiated (64.6%), moderately differentiated (70.2%), and poorly differentiated (85.1%). Conversely, undifferentiated tumours showed lower expression of Ki-67 compared to the degree of differentiation mentioned above.

We did not find any statistically significant relationship between clinicopathological data and Ki-67 index in CRC for the remaining assessed data.

### 3.3. Overall Survival Curves according to Ki-67 Expressions in CRC Tissues

No statistically significant association was observed for Ki-67 expression in CRC tissues (*P* = 0.321 for CRC, and *P* = 0.213 and *P* = 0.874 for colon cancer and rectal cancer evaluated separately, resp.), as observed in Figure 4.

Relatively to CRC, survival of patients that are negative for Ki-67 is 65.6% with a medium of survival of 65.0 ± 2.8 months after diagnosis, while Ki-67 positive patients present a survival of 62.3% with a medium of survival of 62.1 ± 2.1 months after diagnosis, such as is shown in Table 5.

### 3.4. Ki-67 Expressions in Lymph Node Metastasis Samples

A total of 210 samples were organized into TMAs. Additionally 35 patients, with the diagnosis of CRC but without lymph node metastasis, were also selected for control of protein lymph node expression (stages T1 and T2 N0). Sections were evaluated for immunoexpression and the obtained results are given in Table 2, which summarizes the frequency of Ki-67 expression in “normal” lymph nodes and lymph node metastasis.

**Figure 3:**
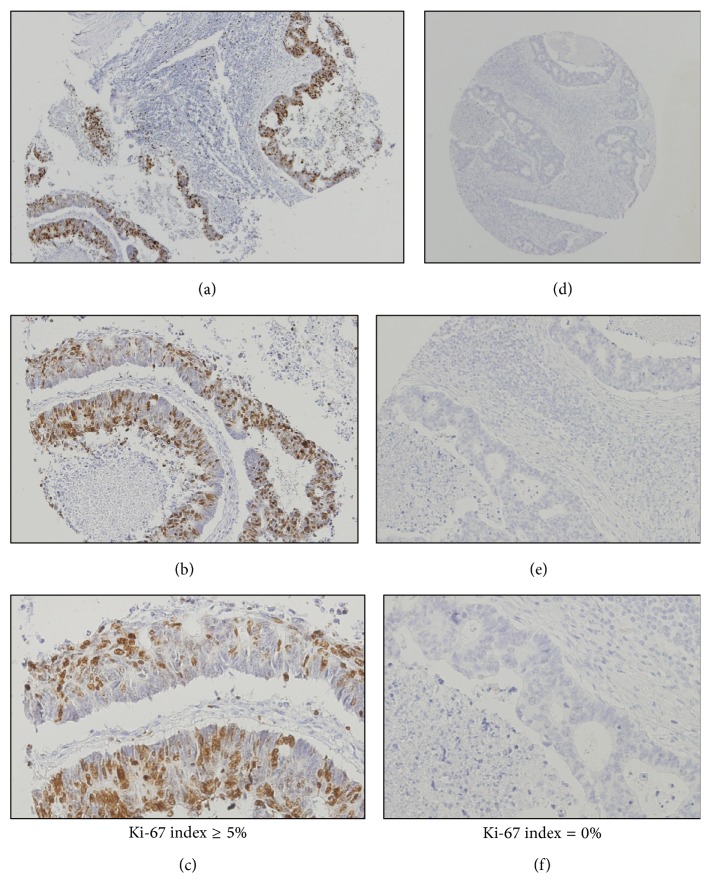
Immunohistochemical expression of Ki-67 in CRC lymph node metastasis samples: ((a) and (d)) original magnification ×40; ((b) and (e)) original magnification ×100; ((c) and (f)) original magnification ×200.

**Figure 4:**
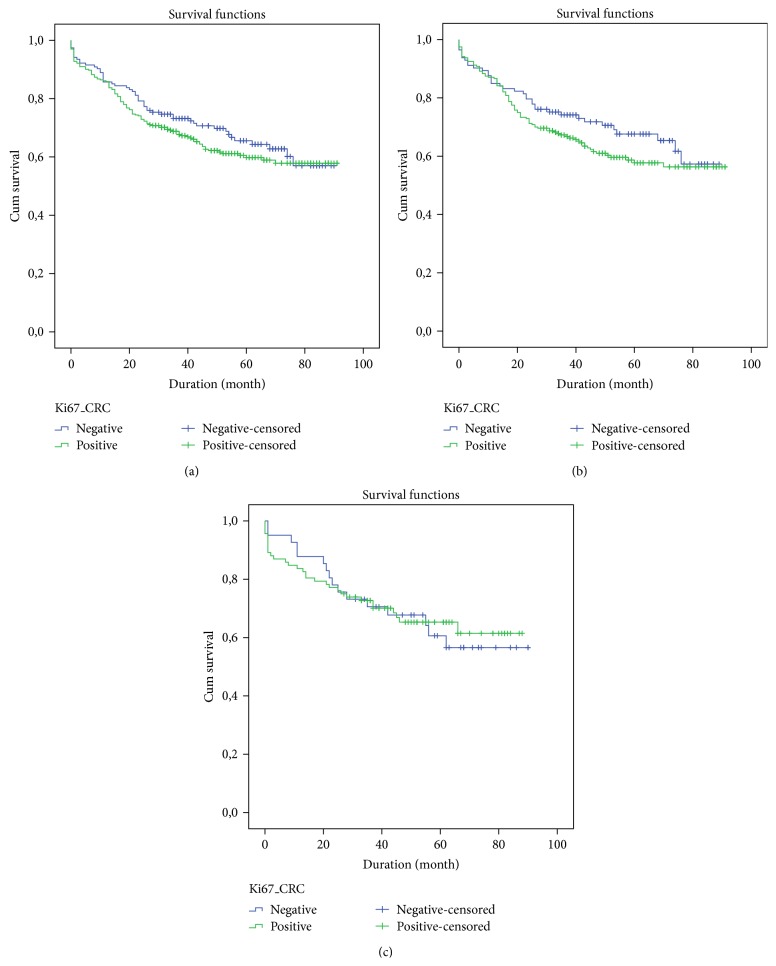
Survival curve of patients with CRC according to Ki-67, assessed by the log-rank test: (a) colorectal cancer: *P* = 0.321; (b) colon cancer: *P* = 0.213; (c) rectal cancer: *P* = 0.874.

We observed that 55.5% (*n* = 60) of the samples of lymph node metastasis were positive for Ki-67, compared to 100% (*n* = 2) of samples of the samples of “normal” lymph nodes. No significant correlation was observed (*P* = 0.502), such as is shown in Table 2 and Figure 3.

### 3.5. Associations between Ki-67 Expressions in Lymph Node Metastasis and Clinicopathological Data

The associations observed between the expression of Ki-67 in lymph node metastasis of CRC and the clinicopathological data are described in Tables 3 and 4. Analyzing these tables, we did not find any statistically significant relationship between clinicopathological data and Ki-67 index in lymph node metastasis.

### 3.6. Overall Survival Curves according to Ki-67 Expressions in Lymph Node Metastasis

Relatively to overall survival, patients with negative Ki-67 index present a survival of 65.3% with a medium of survival of 63.4 ± 5.2 months after diagnosis, while Ki-67 index positive patients present a survival of 51.6% with a medium of survival of 50.0 ± 4.6 months after diagnosis, such as is shown in Table 5.

**Figure 5:**
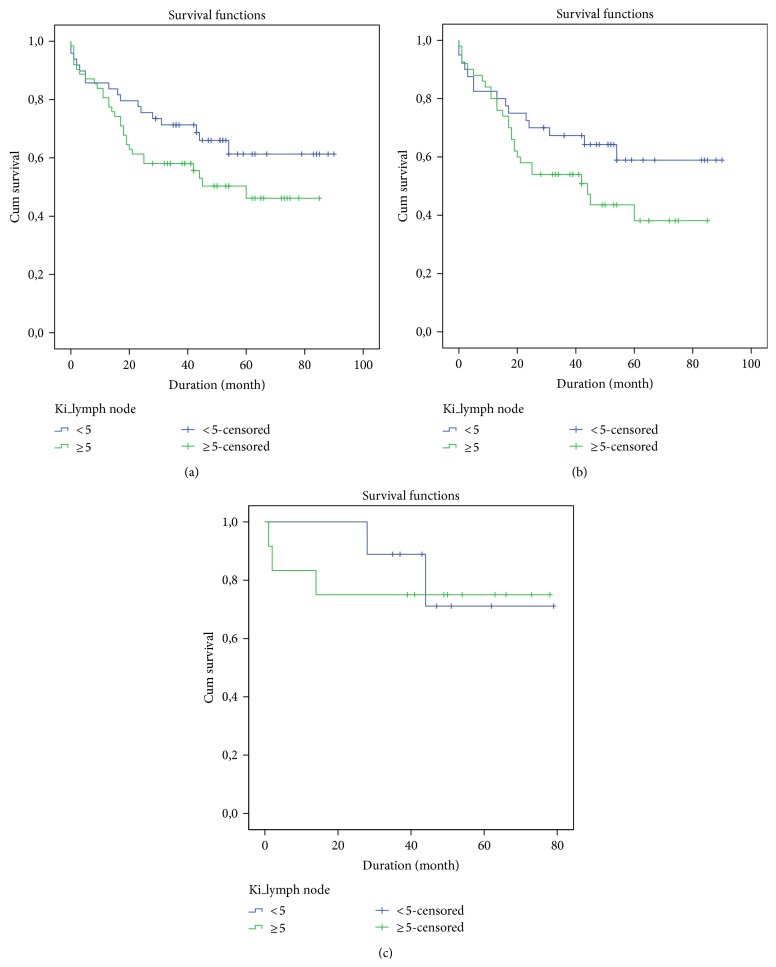
Survival curve of patients with CRC lymph node metastasis according to Ki-67, assessed by the log-rank test: (a) colorectal cancer: *P* = 0.131; (b) colon cancer: *P* = 0.127; (c) rectal cancer: *P* = 0.809.

No statistically significant association was observed for Ki-67 expression in CRC lymph node metastasis tissues (*P* = 0.131 for CRC, *P* = 0.127 and *P* = 0.809 for colon cancer and rectal cancer evaluated separately, resp.); however, a tendency for the relationship between a positive Ki-67 index and a lower overall survival was observed, such as is shown in Figure 5.

### 3.7. Comparing Ki-67 Index Expressions in Lymph Node Metastasis and Primary Tumour

**Figure 6:**
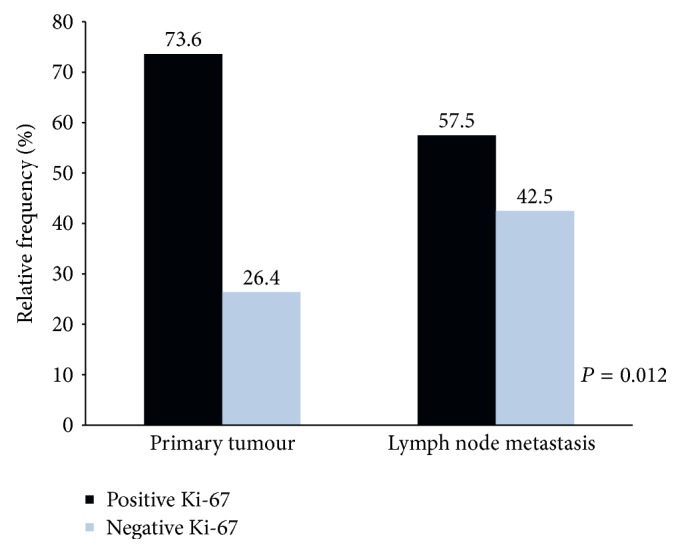
Graphic representation of Ki-67 index in primary CRC tumour and respective lymph node metastasis.

Table 6 represents a comparison between Ki-67 index in the primary tumour and the respective lymph node metastasis. Analyzing this table, it appears that 12 of the cases with negative Ki-67 index in the primary tumour have positive index in lymph node metastasis and that 29 of those with positive index in the primary tumour have a negative index in lymph node metastasis, for a total of 41 discordant cases. This means that there is a significant difference (*P* = 0.012) between the index of Ki-67 in primary tumour and the respective lymph node metastasis. A smaller number of cases of positive Ki-67 index were also observed in lymph node metastasis (*n* = 61; 57.5%) than in the primary tumour (*n* = 78; 73.6%) as is schematized in Figure 6.

## 4. Discussion

The mechanisms that culminate in CRC development, growth, and metastization are still not fully understood. However, common to all cancers are the loss of cellular differentiation and the imbalance between proliferation and cell death; these processes, involved in carcinogenesis, due to its significance, are increasingly being targeted for study.

Ki-67 protein has been widely used as a marker of tumour proliferation [21, 48, 49], and several studies compare Ki-67 index with clinicopathological data and follow-up in CCR [22, 32, 35–40]. With regard to Ki-67 index in lymph node metastasis, as far as we know, this is the first study realized in CRC and, the more similar that we found in literature is the study of Yamauchi et al. [41], which compares Ki-67 index in primary CRC tumours with the respective nodules of peritoneal metastization.

In this study, we determine immunohistochemical expression of Ki-67 protein in CRC samples and respective lymph node metastasis and intended to evaluate possible associations between these expressions and several clinicopathological parameters and patient survival. Further comparison was performed between Ki-67 index in CRC and respective lymph nodes metastasis.

Regarding Ki-67 expression in CRC samples and NA^E^ we observed a significant expression of Ki-67 in the first over the second (*P* < 0.001). These results were expected, since due to its role as a marker of cellular proliferation, a higher expression was expected in tumour tissue than in normal epithelium. These results were also demonstrated by Lin et al. [25].

The same analysis was made for lymph nodes metastasis and “normal” lymph nodes, but no significant correlation was observed (*P* = 0.502). Possible explanations are the fact that “normal” lymph nodes are not truly normal, but of patients with CRC without lymph nodes metastasis (T1 and T2/N0) so this is a bias to be considered since they may already be under the influence of the tumour environment. Another fact that may influence this result is the small size sample of the “normal” lymph node, so further studies need to be realized with bigger samples and normal lymph nodes for control.

When analyzing the correlation of Ki-67 expression in CRC with pathological data, we found an association between the expression of Ki-67 and “tumour penetration” (*P* = 0.013) and “tumour differentiation” (*P* = 0.049).

For “tumour penetration,” we observed a decreasing expression of Ki-67 from the pT1 (79.3%) to pT3 (68.1%) tumours and then a rise in expression for adenocarcinoma with invasion of other organs or structures (pT4) (73.1%).

The decreased expression of Ki-67 with the increasing tumour penetration is conflicting since we would expect an increase in expression with increasing tumour wall penetration, as is observed for pT4. But as the Ki-67 is only a marker of cellular proliferation, other factors may influence this outcome.

Regarding “tumour differentiation,” we observed an increasing expression of Ki-67 from the well-differentiated to the undifferentiated tumours; conversely, undifferentiated tumours showed lower expression of Ki-67 compared to the degree of differentiation mentioned above.

As was observed by some authors [38, 50], the more undifferentiated is the tumour, the higher is the rate of cell proliferation and therefore Ki-67 index. This is not consistent with our findings; however, since the lower expression in undifferentiated tumours may be explained by the small size of this sample, further studies with larger series of undifferentiated tumours are necessary.

When analyzing the correlation of Ki-67 expression in lymph node metastasis with pathological data, any statistically significant relationship was observed. In the literature, no other studies realized in lymph node metastasis of CRC were found, and similar results were observed by Jansson and Sun [39] on the primary tumour but contradict the other studies analyzed [22, 25, 28–30, 32, 35–40].

In our series, we have not observed association between Ki-67 expression and patient's survival, for CRC (*P* = 0.321) and for lymph node metastasis (*P* = 0.131), series and the same was true when we considered separately colon cancer and rectal cancer. This was corroborated by the observations of Jansson and Sun [39]; however, these findings contradict, in part, the report of Valera et al. [35] that studied primary CRC tumours, and also contradict studies made with lymph node metastasis from breast cancer [43–45] and prostate cancer [51], where a higher ki-67 index was associated with a worse patient survival.

Finally, we found association (*P* = 0.012) between Ki-67 index in primary tumour and the respective lymph node metastasis and also observed that Ki-67 index was more often positive in the primary tumour than in the respective lymph node metastasis. This result is consistent with the study carried out by Yamauchi et al. [41] to compare Ki-67 index in CRC primary tumour and respective nodules of peritoneal dissemination, which, as in the present study, present a greater proportion of proliferating cells in the primary tumour than in the nodules of peritoneal dissemination, not advancing; however, there is no explanation for this finding. Distinct results were observed for similar studies realized in breast cancer [42–44], where lymph node metastasis presents higher ki-67 index than primary tumour, but also no explanation was mentioned. Recently, Jo and colleagues have found a significant difference of higher Ki-67 proliferation in nodal metastasis and primary gastric cancer [52]. Most of the criticism of Ki-67 evaluation is related to the differences of antibodies, slide background, retrieval protocols applied in preparing the immunoreaction, and the scores used to evaluate the significance of Ki-67 proliferation rates. This concern is pertinent and most of the works that evaluate this premise did not reach a consensus. Interesting, automated evaluation of the Ki-67 labelled preparation has been adjudicated as superior than manual analyses. Moreover, subdividing the cases in low and high proliferative rate improve the* kappa* correlation. Besides these advisements, the lack of standard protocols among the laboratories limits the clinical relevance of the works [53].

This difference between Ki-67 index in primary CRC tumour and respective lymph node metastasis may explain the absence of correlation with clinicopathological data and survival observed in this study as it shows that the proliferative profile of the primary CRC tumour is different from that of its metastasis. One possible explanation for the low proliferative index of lymph node metastasis as compared to primary tumour is that lymph node cannot represent an optimal proliferative environment, since it is known that nutrient and oxygen deprivation induces cell cycle arrest, leading to a decreased proliferation rate [54]. Another possible explanation may be due to of Ki-67 own usage as a marker of cell proliferation, since Ki-67 expression seems to be influenced by nutrient intake of the cell [48].

The smaller number of patients with a positive Ki-67 index in lymph node metastasis can also contribute to the poor prognosis attributed to the presence of lymph node metastasis [32, 50, 55, 56] in CCR, since most antineoplastic agents target proliferating cells, cells with a low proliferation rate are more resistant to such treatment [53], and this hypothesis was also stated by Cabibi et al. [44], relatively to the subgroup of patients with breast cancer and lower ki-67 index in lymph node metastasis than in primary tumour.

## 5. Conclusions

In this study, we evaluated the immunohistochemical expression of Ki-67 in CRC and respective lymph node metastasis and simultaneously try to determine its correlation with clinicopathological data and patient survival. From the results obtained, it was found that this protein has higher expression in tumour tissue, supporting the hypothesis of involvement of Ki-67 in CRC and its role as a proliferative marker. Furthermore, in CRC samples, the association between the expression of this protein and the degree of tumour differentiation and penetration was found which enables Ki-67 to be used as a potential prognostic factor in CRC.

Although we have not obtained statistical significant results for lymph node metastasis series we observed a statistically significant difference between the Ki-67 index of the primary tumour and the respective lymph node metastasis, which is more often positive in the primary tumour. These results show that lymph node metastasis is composed of proliferating cells slower than that present in the primary tumour and it is hypothesized that the lymph node might not constitute an optimal environment for tumour cells proliferation or that Ki-67 might not be the more suitable proliferative marker for use in lymph node metastasis. This result also raises the possibility that tumour cells in lymph nodes can be more resistant to chemotherapy treatments, thus contributing to the poor prognosis of these patients.

## Figures and Tables

**Table 1:** Detailed aspects of the immunohistochemical procedure used to visualise the Ki-67.

Protein marker	Antigen retrieval	Peroxidase inactivation	Detection system	Antibody		
				Company	Dilution	Incubation period
Ki-67	Citrate Buffer 0.01 M pH = 6.0	3% H_2_O_2_ in methanol, 10 min.	RTU Vectastain ABC elite Reagent	GenNova	1 : 200	Overnight

**Table 2:** Pattern of protein staining in tumour versus normal adjacent epithelium.

Protein marker	Immunoreaction		
Ki-67	*n*	Positive *n* (%)	*P*
CCR			
NA^E^	140	34 (24.3)	**<0.001**
Tumour	506	345 (68.2)	
Lymph node			
Normal	2	2 (100.0)	0.502^*^
Metastasis	109	60 (55.0)	

NA^E^: normal adjacent epithelium;*n*: total number of cases with and without expression of Ki-67; positive *n* (%): total number of cases with expression of Ki-67 and respective percentage.

^*^Comparisons were examined for statistical significance using Fisher's exact test (when *n* < 5).

**Table 3:** Assessment of correlation between Ki-67 expression and clinical data.

	Ki-67 in CRC			Ki-67 in lymph node metastasis		
	*n*	Positive *n* (%)	*P*	*n*	Positive *n* (%)	*P*
Gender						
Male	307	212 (69.1)	0.675	71	41 (57.7)	0.438
Female	180	121 (67.2)		38	19 (50.0)	
Age						
≤45	23	14 (60.9)	0.491	7	4 (57.1)	1.000^*^
>45	464	319 (68.8)		102	56 (54.9)	
Presentation						
Asymptomatic	88	63 (71.6)	0.474	19	8 (42.1)	0.212
Symptomatic	399	270 (67.7)		90	52 (57.8)	
Localization						
Colon	353	240 (68.0)	0.764	90	50 (55.5)	0.841
Rectum	134	93 (69.4)		19	10 (52.6)	
Macroscopic cancer type						
Polypoid	253	171 (67.6)	0.178	45	20 (44.4)	0.373
Ulcerative	111	76 (68.5)		31	20 (64.5)	
Infiltrative	38	22 (57.9)		11	6 (54.5)	
Exophytic	39	32 (82.1)		12	7 (58.3)	
Vilosous	2	2 (100.0)		1	0 (0)	
CEA (ng/mL)						
≤10	337	229 (68.0)	0.750	67	34 (50.7)	0.757
>10	73	51 (69.1)		22	12 (54.5)	

^*∗*^Comparisons were examined for statistical significance using Fisher's exact test (when *n* < 5).

**Table 4:** Assessment of correlation between Ki-67 expression and pathological data.

	Ki-67 in CRC			Ki-67 in lymph node metastasis		
	*n*	Positive *n* (%)	*P*	*n*	Positive *n* (%)	*P*
Tumor size						
≤4.5 cm	279	190 (68.1)	0.519	65	39 (60.0)	0.211
>4.5 cm	179	127 (70.9)		40	19 (47.5)	
Histological type						
Adenocarcinoma	409	281 (68.7)	0.665	88	46 (52.3)	0.483
Mucinous adenocarcinoma	50	32 (64.0)		13	8 (61.5)	
Invasive adenocarcinoma	24	18 (75.0)		6	4 (66.7)	
Signet ring and mucinous	4	2 (50.0)		2	2 (100.0)	
Differentiation						
Well differentiated	209	135 (64.6)	**0.049**	38	19 (50.0)	0.670
Moderately differentiated	208	146 (70.2)		46	26 (56.5)	
Poorly differentiated	47	40 (85.1)		23	14 (60.9)	
Undifferentiated	4	3 (75.0)		1	1 (100.0)	
Tumour penetration						
pT1	34	23 (79.3)	**0.013**	2	2 (100.0)	0.553
pT2	57	39 (68.4)		4	2 (50.0)	
pT3	370	252 (68.1)		96	53 (55.2)	
pT4	26	19 (73.1)		7	3 (42.9)	
Spread to lymph nodes						
Absent	275	188 (68.4)	0.940	9	5 (55.6)	1.000^*^
Present	198	136 (68.7)		89	50 (56.2)	
Venous vessel invasion						
Absent	264	179 (67.8)	0.511	29	13 (44.8)	0.110
Present	201	142 (70.4)		74	46 (62.2)	
TNM						
Stage I	75	54 (72.0)	0.425			
Stage II	181	121 (66.9)				
Stage III	152	108 (71.1)		78	43 (55.1)	0.978
Stage IV	70	45 (64.3)		31	17 (54.8)	

^*∗*^Comparisons were examined for statistical significance using Fisher's exact test (when *n* ≤ 5).

**Table 5:** Survival analysis: frequency and relative frequency for overall survival and medium of time to death.

Ki-67	Tissue	*n*	Deaths *n* (%)	Median for survival time [95% confidence interval]	Log-rank test *P*
Negative	CRC	154	53 (65.6)	65.00 [59.49–70.52]	0.321
Positive		332	125 (62.3)	62.09 [58.06–66.12]	

Negative	Lymph node	49	17 (65.3)	63.48 [53.18–73.79]	0.131
Positive		62	30 (51.6)	50.07 [41.06–59.08]	

**Table 6:** Comparison between Ki-67 index in primary tumor and respective lymph node metastasis.

Ki-67 index in CRC	Ki-67 index in lymph node		
	Negative	Positive	Total *n* (%)
Negative	16	12	28 (26.4)
Positive	29	49	78 (73.6)

Total *n* (%)	45 (42.5)	51 (57.5)	106 (100)

Comparisons were examined for statistical significance using McNemar test.
